# Clinical Features, Diagnosis and Management of Patients with Suspicion of Fascioliasis in Kohgiluyeh and Boyer-Ahmad Province, Southwestern Iran

**Published:** 2020

**Authors:** Abdolali MOSHFE, Arash ARIA, Najme ERFANI, Ali JAMSHIDI, Bahador SARKARI, Samaneh ABDOLAHI KHABISI, Nasir AREFKHAH

**Affiliations:** 1. Cellular and Molecular Research Center, Yasuj University of Medical Sciences, Yasuj, Iran; 2. Department of Parasitology and Mycology, School of Medicine, Shiraz University of Medical Sciences, Shiraz, Iran; 3. Basic Sciences in Infectious Diseases Research Center, Shiraz University of Medical Sciences, Shiraz, Iran; 4. Department of Parasitology and Mycology, School of Medicine, Zahedan University of Medical Sciences, Zahedan, Iran

**Keywords:** Clinical features, Diagnosis, Treatment, Fasciolosis, Iran

## Abstract

**Background:**

In the current study, we described the epidemiological features, clinical presentation, diagnosis and management of patients with suspicion of fascioliasis in Kohgiluyeh and Boyer-Ahmad Province in southwest of Iran.

**Methods:**

Overall, 56 patients with suspicion of fascioliasis, based on their clinical signs and symptoms that referred to Clinic of Internal Medicine in Yasuj city, from 2014 to 2016 were enrolled. Demographic data, history of eating aquatic local plants, the chief complains, and laboratory findings were recorded for each patient. Stool samples were obtained from each case for detection of *Fasciola* eggs. Moreover, blood samples were taken from each patient and evaluated for detection of anti-*Fasciola* antibodies by an indirect ELISA. Patients who defined as having fascioliasis were treated with triclabendazole and were followed for at least three months for clinical improvement.

**Results:**

Serological test was positive in 5 patients. Of these 5 cases, three cases had a history of ingesting raw aquatic vegetables. The main clinical signs and symptoms in positive cases were; abdominal pain (60%), epigastric pain (40%), anemia (60%), and dermal pruritus (20%). Hypereosinophilia was seen in all of 5 positive cases. No *Fasciola* egg was found in stool specimens of any of the patients. The fascioliasis cases were treated by triclabendazole and clinical symptoms disappeared in all of 5 cases.

**Conclusion:**

Our observation further confirmed Yasuj district as a human endemic area for fascioliasis in Iran. The study also highlighted the importance of clinical features together with eosinophilia, as key parameters, in the diagnosis of human fascioliasis. Clinicians need to be aware of this disease and should keep in mind fascioliasis when hypereosinophilia present in patients in such endemic areas.

## Introduction

Fascioliasis caused by liver flukes *Fasciola hepatica* and *F. gigantica* via ingestion of aquatic plants contaminated with encysted metacercariae stage of the worm (1). Although human fascioliasis is frequently reported from Andean and some of European countries, the highest prevalence of human fascioliasis with relatively high annual cases have been reported from a few countries, including Egypt, Iran, Peru and Bolivia (1–5).

Human fascioliasis has two distinct clinical phases; the time of migration of juvenile fluke and when the adult worm get into the bile duct and settle. A symptomless incubation period, lasting for a few days to a few months, starts once the larvae are ingested with contaminated aquatic plants and followed by an acute and a chronic clinical phase. The acute phase starts when the immature worms are migrating through the liver. The juvenile flukes puncture the liver’s surface and move around until they reach the bile ducts. This invasion is accompanied by a swollen liver, skin rashes and extreme abdominal pain (6, 7). The chronic phase begins when the worms reach the bile ducts, which cause intermittent pain, cholangitis, obstructive jaundice, and eosinophilia (6). Diagnosis of fascioliasis relies on its clinical features along with laboratory methods (8–12).

Previous studies demonstrated a new focus of human fascioliasis in Yasuj district in Kohgiluyeh and Boyerahmad Province, southwest of Iran, where animal fasciolosis is quite common (4, 5, 13, 14). Both *F. hepatica* and *F. gigantica* are present in animals in this area (13–15). Human infection has been confirmed in some patients by parasitological (detection of eggs in stool samples), serological (ELISA and western blotting) and molecular (PCR and sequencing) approaches in this area (4, 5, 8). Molecular studies demonstrated *F. hepatica* as the causative agent of human fascioliasis in few of the patients in the area (4).

Nowadays, physician awareness about human fascioliasis in this human endemic area of fascioliasis has increased and during the last 10 years, progress has been made in understanding the clinical features of fascioliasis. Therefore, much more cases with suspicion of fascioliasis have been referred to the university-affiliated health centers for proper diagnosis and management by general practitioners, infectious disease specialist as well as internist.

Both ELISA and western blotting have been used for diagnosis of suspected cases in the area. The sensitivity and specificity of serological methods, using ELISA based on *Fasciola* excretory-secretory (ES) antigens exceed 95% (12). Results of serological testing may become positive 2–4 weeks after infection, preceding the presence of eggs in the stool.

Eosinophilia is more likely to be present during the parenchymal phase; however, the eosinophil count may be normal in up to 50% of chronic cases. Normal eosinophil count cannot be used to exclude parasitic etiology (16). On the other hand, stool microscopy is not conclusive for the diagnosis of human fascioliasis in the acute phase of illness, as the pre-patent period (time from infection to shedding of ova in the feces by mature adult worms) is around four months (10).

In this study, we described the epidemiological features, clinical presentation, diagnosis and management of patients with suspicion of fascioliasis, misdiagnosed with other diseases, in Kohgiluyeh and Boyer-Ahmad Province, Southwestern Iran. Moreover, we highlighted the importance of clinical presentation and diagnostic parameters of fascioliasis, underlined the significant role of eosinophilia in the diagnosis of human fascioliasis.

## Materials and Methods

Overall, 56 patients with suspicion of fascioliasis, based on their clinical signs and symptoms that referred to Clinic of Internal Medicine, a university-affiliated clinic, in Yasuj City, Southwestern Iran during Feb 2014 to Sep 2016 were enrolled in this study. While patients were being seen by the internist, demographical data, history of eating aquatic local plants, the chief complains, clinical presentation and the available laboratory findings (mainly eosinophilia) were recorded. Stool samples were obtained from each case in three subsequent times for detection of *Fasciola* spp. eggs. Moreover, 5 mL of fresh blood was taken from each patient for serological evaluation of fascioliasis. Each stool sample was examined by concentration method (Formalin Ether). Sera samples were sent to the Department of Parasitology and Mycology, at School of Medicine in Shiraz University of Medical Sciences (SUMS) for detection of anti-*Fasciola* antibodies by an indirect ELISA, using excretory/secretory antigens of *F. hepatica* (8, 17). Patients who defined as having fascioliasis, based on a positive serological test and conclusive signs and symptoms, were treated with triclabendazole (2 individual doses of 10 mg/kg, separated in time by 24 h). Treated patients were followed for at least 3 months for recovery and improvement of the symptoms. Criteria for improvement were disappearance of main symptoms of fascioliasis and reduction in blood eosinophilia (less than 450/mm).

The study was approved by Ethical Committee of Yasuj University of Medical Sciences (YUMS) and informed consent was obtained from each participant before enrolling in the study.

All the collected data were analyzed, using SPSS software (ver. 18) (Chicago, IL, USA).

## Results

Of 56 clinical suspicion cases of fascioliasis, 5 (8.9%) female patients were positive by ELISA test. Of these cases, 4 patients were above 50 yr old and 3 cases had a history of ingesting raw aquatic vegetables (locally named, Bakaloo). The main clinical signs and symptoms in positive cases were; abdominal pain in 3, epigastric pain in 2, anemia in 3 cases and dermal pruritus and nausea each in 1 patient. Hypereosinophilia was seen in all of 5 positive cases. No *Fasciola* eggs were found in stool specimens of any of the patients. Obstructive jaundice was not seen in any of the positive cases. The positive cases were treated by triclabendazole and clinical symptoms disappeared in all of 5 cases when followed for 3 months after treatment. Table 1 shows the age distribution of patients’ suspicion of fascioliasis.

**Table 1:** Age distribution of patients’ suspicion of fascioliasis in Yasuj district, southwest of Iran

	***Serological assay (ELISA)***					
	**Negative**		**Positive**		***Total***	
***Age (yr)***	**No.**	**Percent**	**No.**	**Percent**	**No.**	**Percent**
≤ 10	8	14.3	0	0	8	14.3
11–20	7	12.5	0	0	7	12.5
21–30	11	19.6	1	1.8	12	21.4
31–40	6	10.7	0	0	6	10.7
41–50	11	19.6	0	0	11	19.6
51–60	5	8.9	1	1.8	6	10.7
≥ 60	3	5.4	3	5.4	6	10.8
Total	51	91.1	5	8.9	56	100

## Discussion

Fascioliasis is an important helminth infection caused by *F. hepatica* and *F. gigantica* (1). Human is an accidental host and can be infected through ingestion of metacercaria-carrying aquatic plants or contaminated water. The main clinical manifestation of human fascioliasis are the abdominal pain mainly in right upper quadrant, nausea, vomiting, skin rash, itching, obstructive jaundice, cholangitis, and hypereosinophilia. Clinical suspicion of fascioliasis may arise in patients complaining of right upper quadrant pain, fever, anorexia, and jaundice. Moreover, the presence of eosinophilia along with a history of ingestion of aquatic plants in endemic areas may lead to the suspicion of fascioliasis.

Human fascioliasis is a considerable health problem in Iran, especially in the north of the country where two biggest outbreaks of human fascioliasis in the world have occurred during the last decades (2, 3).

Yasuj is a mountainous district in the southwest of Iran, which has recently been introduced as a new focus of human fascioliasis (4, 5). The unique climate condition of this region favored the spread of numerous parasitic diseases (4, 5, 18–20). In this area, fascioliasis is probably transmitted to human by the use of freshwater plants such as *Nasturtium microphyllum* (local name, Bakaloo) and *Menthe logifolia* (local name, Pooneh). The disease is also quite common in livestock in the area (13, 14, 21). Both *F. hepatica* and *F. gigantica* have been isolated from animals in the region while the species responsible for human fascioliasis is reported to be *F. hepatica* (4, 5, 13).

In the current study fascioliasis patients were complaining of abdominal pain (40%), itching (20%) and nausea (20%). Similar clinical features have been reported in a familial outbreak of 24 fascioliasis patients in eastern Anatolia, in Turkey (22).

In our study, *Fasciola* eggs were not found in the stool specimens of any of the patients. The absence of eggs in the stool samples of positive cases may be due to the inability of *Fasciola* to produce eggs, lack of adaptation of the fluke to the human host, or encapsulation of eggs in the liver granuloma, and low egg releasing because of low infection burden or old infection (12). Furthermore, intermittent egg production and cessation of egg shedding in the advanced chronic phase of fascioliasis is common. Failure to find eggs in the patient’s stools may also be due to the disease being in the acute phase. Besides, the biliary obstruction can also be one of the causes of the lack of eggs in the patient’s stool sample. In keeping with our findings, none of the serologically proven fascioliasis cases in an outbreak of human fascioliasis in Kermanshah in the western part of Iran was egg positive (23). Similar findings have been described in a case series of fascioliasis in Australian travelers to Bali, where 6 cases of fascioliasis have been reported and stool microscopy have been negative in all of 6 cases (24). These observations are consistent with our findings. In a study of 711 fascioliasis cases in France during a 30-year epidemiological survey, only 27.6% of cases were egg-positive (25). Moreover, in a series of 23 cases of serologically and clinically proven human fascioliasis in Egypt, only two cases (8.7%) were egg positive (26).

Serological diagnosis, based on antibody detection, is an appropriate approach for the diagnosis of fascioliasis. This is mainly because the patient usually presents clinical signs or symptoms long before than the egg appears in the stool, whereas antibodies to *Fasciola* antigens can be detected in patient’s sera two weeks after infection, long before the beginning of egg shedding (10). In our study, an ELISA system, based on ES antigens of *F. hepatica*, was applied for the serological diagnosis of fascioliasis cases. This ELISA system has appropriate performance (more than 95% sensitivity and specificity), in the diagnosis of human fascioliasis (12).

Marked eosinophilia is one of the hallmarks of acute fascioliasis and in the endemic areas; it should raise the suspicion of fascioliasis. In our cases, blood eosinophilic counts were considerably high in all of the 5 patients. Eosinophilia is a key feature of fascioliasis, which is present in most of the fascioliasis patients. Eosinophilia can be seen during parenchymal phase as well as in the chronic phase (24). In the case series of 24 fascioliasis patients in Anatolia, eosinophilia was seen in 70% of the cases. The authors concluded that eosinophilia is an important predictor for fascioliasis in the patient with consistent clinical manifestations (22). Eosinophilia disappears after successful treatment and if remains it can be a clue for treatment failure.

Triclabendazole is considered the drug of choice for treatment of human fascioliasis. It is a benzimidazole derivative with a cure rate of around 80% with the first course and almost 90–100% with the second course of treatment. The drug has been successfully used for the treatment of human fascioliasis in the two major outbreaks of fascioliasis in Iran (3). In our study, clinical improvement was seen in all of the fascioliasis patients when treated with a double dose of triclabendazole.

Taken together, our observation further confirmed Yasuj district as a human endemic area for fascioliasis in Iran. The study also highlighted the importance of clinical features together with eosinophilia and a positive serological test as key parameters in the diagnosis of human fascioliasis in such endemic areas. Clinicians need to be aware of this disease and should keep in mind fascioliasis when dominant clinical presentations along with hypereosinophilia present in patients in endemic areas of fascioliasis.

Having said that, physicians working in the region should not attribute any abdominal pain with eosinophilia to fascioliasis, as eosinophilic patients with abdominal pain may be infected with other parasitic helminths such as *Trichostrongylus*, which may also be present in that area (27).

## Conclusion

Yasuj district should be regarded as a human endemic area for fascioliasis in Iran. We highlighted the importance of clinical features together with eosinophilia, as key parameters, in the diagnosis of human fascioliasis. Clinicians need to be aware of this disease and should keep in mind fascioliasis when hypereosinophilia present in patients in such endemic areas.
